# Validation of EuroSCORE II Scoring System on Isolated CABG Patient in Indonesia

**DOI:** 10.1186/s43044-023-00410-0

**Published:** 2023-10-13

**Authors:** Rita Zahara, Daondy Friarsa Soeharto, Bambang Widyantoro, Sugisman, Bagus Herlambang

**Affiliations:** 1 Department of Cardiology and Vascular Medicine, Faculty of Medicine, Universitas Indonesia/National Cardiovascular Center Harapan Kita, Jakarta, Indonesia; 2 National Cardiovascular Center Harapan Kita, Jakarta, Indonesia; 3 Department of Thoracic, Cardiac and Vascular Surgery Faculty of Medicine, Universitas Indonesia/National Cardiovascular Center Harapan Kita, Jakarta, Indonesia

**Keywords:** EuroSCORE II, Isolated CABG, Validation, Mortality, Indonesia

## Abstract

**Background:**

Coronary Artery Bypass Graft (CABG) is one solution to overcome cardiovascular problems. EuroSCORE II is a scoring system to predict mortality risk in patients undergoing cardiac surgery including CABG. Unfortunately, there’s still much debate about the benefits of EuroSCORE II in Asia, including Indonesia. This study aims to validates EuroSCORE II in predicting the outcomes in patients underwent CABG without any other procedure.

**Results:**

A total of 2628 patients were included. The mean age was 59 years, mostly male (84.97%; *n* = 2233). Most patients underwent elective surgery (93.07%; *n* = 2446) and only 1.67% (*n* = 44) of the patients has high EuroSCORE category. Death was found in 4.22% (*n*-111) patients. EuroSCORE II had fair discriminant power (AUC 0.72), but a lower mortality predicted value for each group.

**Conclusion:**

The parameters in EuroSCORE II are related with mortality in isolated CABG patients, but they cannot be used as mortality predictors in Indonesia.

## Background

Cardiovascular disease is one of many health problems that causes significant death rate in the world. Around 31% of deaths are caused by cardiovascular disease worldwide, and in Indonesia it was one of three most death-causing non communicable diseases, besides stroke and cancer [1]. One of the solutions provided to overcome this problem is *Coronary Artery Bypass Graft* (CABG). This procedure is mainly indicated for patients who are at high risk of death, or have more than one vascular problem, or myocardial infarction that cannot be managed by Percutaneous Coronary Intervention (PCI) [2]. Although this procedure can reduce mortality, there is still a chance that the patient will have a poor outcome. European System for Cardiac Operative Risk Evaluation (EuroSCORE) is a scoring system to predict mortality risk in patients undergoing cardiac surgery, including CABG. This scoring system was published for the first time in 1999, and then updated in 2012 [3].

Several follow-up studies have been done to validate EuroSCORE II around the globe. Noyez, et.al stated that the parameters used in EuroSCORE II can indeed reduce the chance of over estimation of mortality risk that occurred in the previous scoring system [4]. Other meta-analysis studies conducted in the United States and England also provided the same conclusion, i.e., that EuroSCORE II have a higher predictive value and overpowering ACEF scoring system in terms of in-hospital mortality and 30-day mortality [5].

Unfortunately, there’s still much debate about the benefits of EuroSCORE II in Asia, including Indonesia. A study in Kuala Lumpur showed that EuroSCORE II is suitable enough to predict mortality after CABG procedures, but in India, EuroSCORE II was shown to have bad discrimination power and poor calibration [6, 7]. In Indonesia, EuroSCORE II is yet to be used as consideration for managing patients undergoing major cardiac surgery due to the minimum number of supporting studies. A cohort study conducted in Surabaya showed that EuroSCORE II has fair calibration and discrimination power, but recently another study also conducted in Surabaya stated that EuroSCORE II is less effective for use in Indonesia [8, 9]. A recent multi-center study also supports the statement that EuroSCORE II is a poor predictor for major cardiac surgery patient outcomes in Indonesia [10]. Isolated CABG is a cardiac procedure in which only CABG is performed without any other procedure such as valve repair, structural repair, aorta surgery, or tumor resection. Currently, there are no studies that focus on analyzing the benefit of EuroSCORE II in isolated CABG patient populations in Indonesia.

## Methods

This research is a cross sectional retrospective study conducted at the Harapan Kita National Heart Center. Ethical clearance and research approval were granted by the director of Harapan Kita National Heart Center. Variables’ definitions such as COPD, poor mobility, endocarditis, previous cardiac surgery, kidney failure category, ejection fraction category, and procedural status were based on the original EuroSCORE II study. Subjects were taken by total sampling. All patients aged 18 years or older who underwent an isolated CABG procedure between January 2017 and June 2022 and had sufficient data for EuroSCORE II calculation were included in this study. Patient data were extracted from the registry of Adult Surgery Division, Research and Development Installation of Harapan Kita National Heart Center.

Data processing was performed using SPSS 17 software. Univariate analysis was performed on patient characteristic data. Categorical data are displayed in proportion or percentage, and numerical data in the form of mean and standard deviation (if normally distributed) or median and minimum–maximum (if not normally distributed). Discriminatory power and calibration tests were performed to assess the predictive performance of EuroSCORE II. The area under the receiver operating characteristics (ROC) curve was observed to estimate the discriminatory performance of EuroSCORE II in predicting in-hospital mortality. The Hosmer–Lemeshow goodness-of-fit test was performed to assess calibration.

## Results

From January 2017 to June 2022, 2862 isolated CABG procedures were performed at Harapan Kita National Heart Center, in which a total of 2628 patients meet the inclusion criteria. From the patients included in the study, 75% (*n* = 2175) is in low EuroSCORE II category, 16% (*n* = 409) in moderate category and 2% (*n* = 44) in high category. Complete characteristics can be seen in Table 1. This study found that factors assessed by EuroSCORE II is in fact related with mortality in Indonesian isolated CABG patients as shown in Table 2.

**Table 1:** Sample characteristic

Variable	EuroSCORE II						Total	
	Low (< 2%)		Moderate (2–5%)		High (> 5%)			
	(n)	%	(n)	%	(n)	%	(n)	%
Age	58.08 (± 7.67)		62.67 (± 8.71)		64.20 (± 7.96)		58.90 (± 8.041)	
Body mass index	26.97 (± 12.31)		24.66 (± 3.73)		25.80 (± 3.58)		27.40 (± 37.640	
Sex								
Male	1885	86.67	313	76.53	35	79.5	2233	84.97
Female	290	13.33	96	23.47	9	20.5	395	15.03
Risk factor								
Smoker	1159	53.29	210	51.34	24	54.5	1393	53.01
Family history of CAD	331	15.22	63	15.40	8	18.2	402	15.30
Diabetes	863	39.68	211	51.59	23	52.3	1097	41.74
Insulin use	69	3.17	46	11.25	2	4.55	117	4.45
Dyslipidemia	774	35.59	156	38.14	25	56.8	955	36.34
Kidney failure	113	5.20	66	16.14	9	20.5	188	7.15
Cr clerance > 85	751	34.53	21	5.13	4	9.09	776	29.53
Cr clearance 50–85	1177	54.11	124	30.32	12	27.3	1313	49.96
Cr clearance < 50	226	10.39	251	61.37	28	63.6	505	19.22
Dialysis	21	0.97	13	3.18	0	0	34	1.29
Hypertension	1410	64.83	296	72.37	35	79.5	1741	66.25
Stroke	145	6.67	50	12.22	7	15.9	202	7.69
Endocarditis	0	0.00	0	0.00	0	0	0	0.00
COPD	24	1.10	12	2.93	0	0	36	1.37
Immunosuppressant therapy	12	0.55	2	0.49	0	0	14	0.53
Cerebrovascular disease	75	3.45	31	7.58	4	9.09	110	4.19
Cardiology specific risk factor								
NYHA FC								
I	370	17.01	31	7.58	3	6.82	404	15.37
II	1281	58.90	172	42.05	15	34.1	1468	55.86
III	488	22.44	183	44.74	15	34.1	686	26.10
IV	36	1.66	23	5.62	11	25	70	2.66
CCS = 4	42	1.93	29	7.09	12	27.3	83	3.16
Arrhythmia	3	0.14	27	6.60	18	40.9	48	1.83
Cardiogenic shock	1	0.05	3	0.73	12	27.3	16	0.61
Myocardia infarct	799	36.74	226	55.26	32	72.7	1057	40.22
< 24 Hours	30	1.38	12	2.93	8	18.2	50	1.90
1–7 Days	36	1.66	27	6.60	6	13.6	69	2.63
8–21 Days	41	1.89	23	5.62	4	9.09	68	2.59
> 21 Days	590	27.13	130	31.78	9	20.5	729	27.74
Missing	102	4.69	34	8.31	5	11.4	141	5.37
Left main disease	728	33.47	148	36.19	23	52.3	899	34.21
Ejection fraction	56.02 (± 7.66)		44.74 (± 15.13)		38.68 (± 13.60)		53.98 (± 13.956)	
Good	1533	70.48	135	33.01	6	13.6	1674	63.70
Moderate	581	26.71	186	45.48	26	59.1	793	30.18
Poor	59	2.71	73	17.85	7	15.9	139	5.29
Very poor	2	0.09	15	3.67	5	11.4	22	0.84
Previous cardiac surgery	5	0.23	17	4.16	10	22.7	32	1.22
Poor mobility	232	10.67	116	28.36	23	52.3	371	14.12
Procedural status								
Elective	2084	95.82	336	82.15	26	59.1	2446	93.07
Urgent	90	4.14	71	17.36	16	36.4	177	6.74
Emergency	1	0.05	2	0.49	2	4.55	5	0.19
Mortality	60	2.76	36	8.80	15	34.1	111	4.22

*CAD* coronary artery disease, *Cr* Creatinine, *COPD* Chronic Obstructive Pulmonary Disease, *NYHA FC* New York Heart Association Functional Class, *CCS* Canadian Cardiovascular Society grading of angina

**Table 2:** Association  between risk factor and mortality

Variable	Mortality		P	OR	95% CI
	n	(%)			
*Patient-related factors*					
Sex					
Male	86	3.9	0.02		
Female	25	6.3			
Risk factor					
Diabetes	57	5.2	0.036	1.5	1.025–2.193
Kidney failure	12	6.38	0.127	1.61	0.869–2.992
Cr clearance 50–85	48	3.7	0.148	0.75	0.514–1.107
Cr clearance < 50	37	7.3	< 0.000	2.19	1.457–3.289
Dialysis	4	1.4	0.025	3.25	1.091–9.658
Critical state					
Arrhythmia	6	12.5	0.004	3.37	1.4–8.094
Cardiogenic shock	6	37.5	< 0.000	14.33	5.110–40.159
Poor mobility	35	9.43	< 0.000	2.99	1.971–4.534
Previous cardiac surgery	4	12.5	0.019	3.32	1.145–9.643
*Cardiac related factors*					
NYHA FC					
NYHA I	13	3.2	< 0.000		
NYHA II	50	3.4			
NYHA III	37	5.4			
NYHA IV	11	15.7			
Myocardial infarct	59	5.58	0.005	1.73	1.179–2.528
7–24 h	6	12	< 0.000		
1–7 days	11	16.18			
8–21 days	4	5.88			
> 21 days	28	3.84			
Ejection fraction					
Good	53	3.17	< 0.000		
Moderate	37	4.7			
Poor	16	11.5			
Very poor	5	22.7			
*Operation related factors*					
Procedural status					
Elective	90	3.7	< 0.000		
Urgent	19	10.7			
Emergency	2	40			
Category EuroSCORE II					
Low	60	2.8	< 0.000		
Moderate	36	8.8			
High	29	34.1			

*Cr* Creatinine, *NYHA FC* New York Heart Association Functional Class

**Fig. 1:**
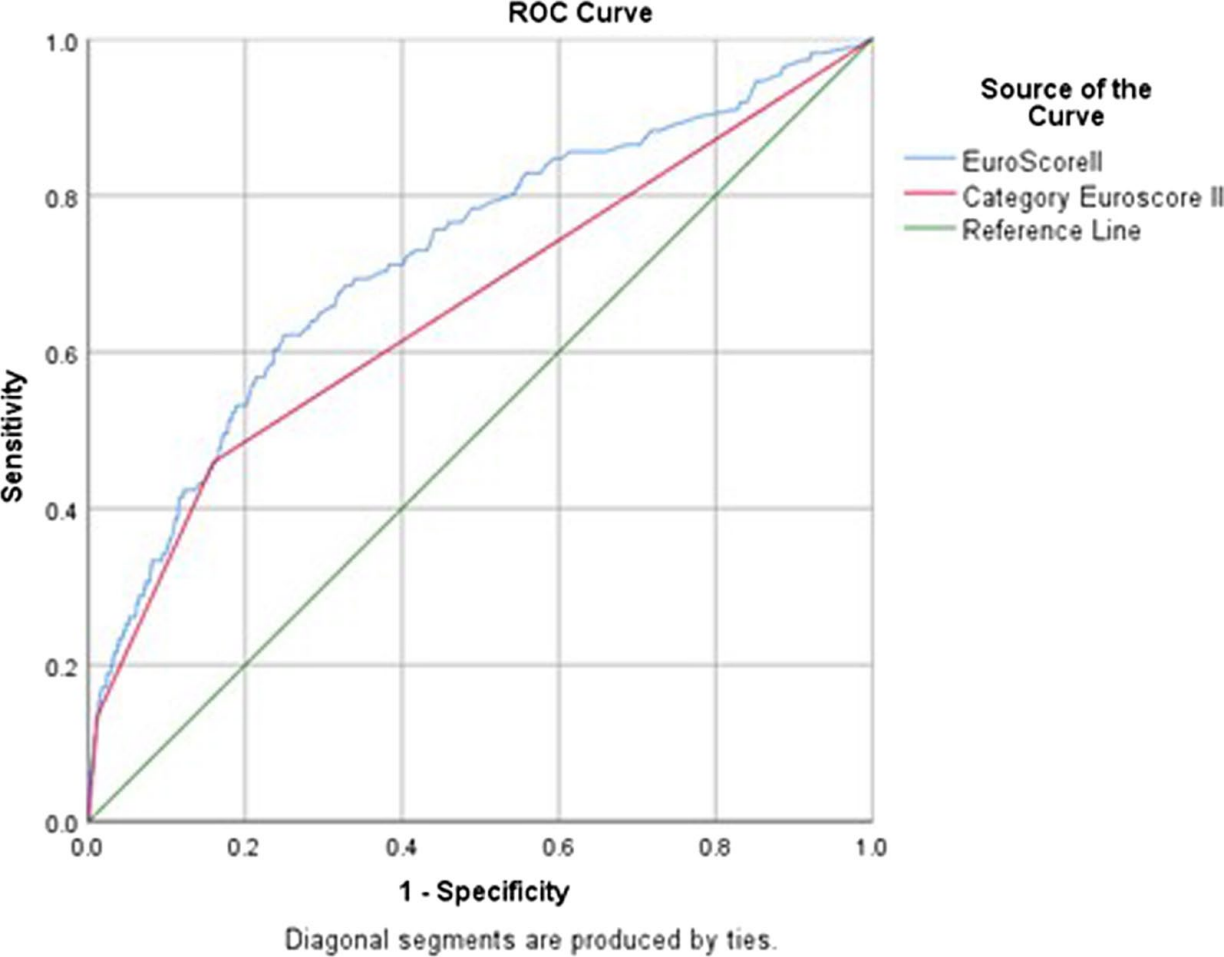
ROC curve of EuroSCORE II discrimination on mortality prediction

**Fig. 2:**
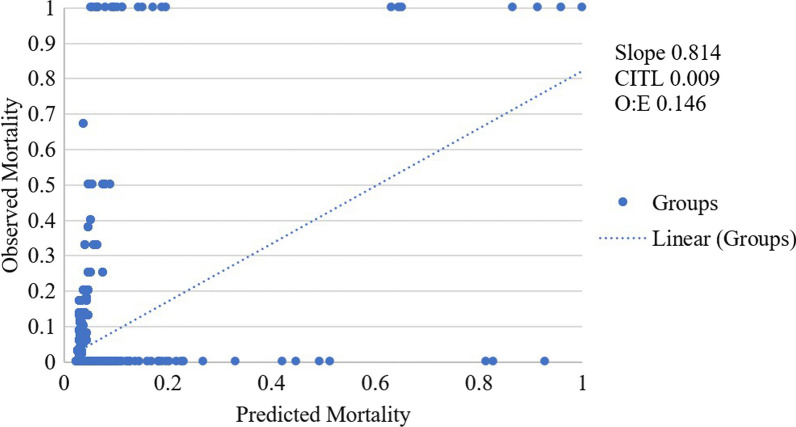
Calibration plot of EuroSCORE II

Based on the receiver operating characteristic (ROC) curve, it can be seen that EuroSCORE II has a fair discriminant power with an area under the curve (AUC) of 0.72 (Fig. 1). However, when reanalyzed with Hosmer–Lemeshow to determine calibration, EuroSCORE II was found to have poor calibration (*p* = 0.02), with a lower mortality predictive value in each risk category group (Table 3). The calibration plot also suggest the same result with slope value of 0.814 and CITL value of 0.009 (Fig. 2).

**Table 3:** Association between predicted mortality and observed mortality in isolated CABG patients

Group	Observed mortality		Predicted mortality		AUC	95% CI	pAUC	Hosmer–lemeshow Chi-square	*p*-HL
	n	(%)	n	(%)					
Total sample	111	4.2	11	0.4	0.72	0.67–0.77	0.00	17.73	0.02
Category EuroSCORE II									
Low	60	2.8	0	0	0.66	0.60–0.71	0.00	5.96	0.65
Moderate	36	8.8	0	0				5.85	0.66
High	15	34.1	7	15.9				8.91	0.35

*AUC* area under the curve, *pAUC P* value of area under the curve, *p-HL P* value of Hosmer–Lemeshow

## Discussion

Despite of fair discrimination power, current study suggests that EuroSCORE II underestimate mortality risk in all scoring category in this population. This result is supported by a multi-center study done by Kurniawaty, et.al conducted at Dr. Sardjito Hospital, Kariadi Hospital, and Abdul Wahab Sjahranie Hospital, which stated that EuroSCORE II had a poor predictive value and led to an underestimation of mortality risk in patients undergoing major cardiac surgery, though some parameters included in the scoring system did have significant association with patient outcomes. Diabetes, history of previous cardiac surgery, left ventricular dysfunction, history of myocardial infarction, and procedure status which each had a significant OR in this study were found to have no significant association in this multi-center study. It should be emphasized that these studies involved several types of cardiac surgical procedures, and the majority were non-coronary procedures [10].

In contrast, Sembiring, et.al found a slightly different results where EuroSCORE II had good calibration as a predictor for mortality in patients undergoing major cardiac surgery (including surgery, heart tumor surgery, aorta surgery, or a combination of these surgeries) based on the Hosmer–Lemeshow analysis (*p* = 0.55), and the area under the ROC curve is 0.85 which indicates a good discriminant value. However, this study actually overestimated mortality in EuroSCORE II. An additional finding obtained from that study was that this scoring system tends to overestimate the risk of death for the group of patients with EuroSCORE II < 1.3% and underestimate the risk of death for the group of patients with EuroSCORE II > 2.3% [8]. Another study that also used samples undergoing isolated CABG in Medan also found a statistically significant association with EuroSCORE II stratification on mortality. Moreover, EuroSCORE with a value of > 3.31 could be used to predict major cardiovascular events after CABG with a sensitivity of 90% and a specificity of 90%. Unfortunately, the study did not validate the accuracy of the use of EuroSCORE II itself [11].

The patient’s overall characteristics in this research were not significantly different from those of the original EuroSCORE II study. The only significant difference between the two studies was that endocarditis was not found in any of the patients in this study [3]. Patients with endocarditis are more likely to undergo concurrent valve surgery procedures; whereas, this study focused on patients undergoing CABG alone. Stroke (OR 2.28; 95% CI 1.331–3.903) and hypertension (OR 1.62; 95% CI 1.039–2.511) were found to significantly increase the risk of mortality in this study. These two comorbidities had not been considered in the original EuroSCORE II study, although Herlitz, et.al already suggested in their study that hypertension increases the risk for direct post-procedural complications and two year mortality rate [3, 12]. The effect of stroke on the increased risk of death in this study may because patients with a history of stroke also have poor mobility. Bottle, et.al found that history of stroke before CABG did not affect the outcomes of the procedure unless accompanied by other morbidities [13]. In this study, the onset of myocardial infarction was also found to influence the mortality risk in patients. In the original EuroSCORE II study, similar results were found with onset category of < 72 h, 72 h—three months, and more than three months. This parameter was not included in the final scoring system because it was considered to have the same value as the patient's procedure status, and when reanalyzed by the regression method, the association of onset with mortality risk was significantly reduced [3].

In Asia, the use of EuroSCORE II as a mortality predictor in post-cardiac surgery patients is still being debated. Studies in Malaysia and Bangladesh that were also conducted in isolated CABG populations found that EuroSCORE II was good enough to predict mortality in their research population. Musa, et.al found no significant difference between predicted mortality based on EuroSCORE II and observed mortality, which indicates a good model calibration [6]. Ranjan et.al who found that EuroSCORE have an important role in predicting early prognosis and end outcome also support the previous study, even though there’s a little discrepancy between the expected mortality and the observed mortality in their result [14]. In India, EuroSCORE II is considered not ideal for predicting mortality in post-major cardiac surgery patients according to a cohort study which found that the scoring system only correctly predicted the low and moderate risk patients, but overestimated the high risk group [7].

Research conducted in Greece, Serbia, the Netherlands, Argentina, as well as meta-analysis studies in America and the UK suggest that EuroSCORE II has improved the previous scoring system and is quite valid to predict mortality in post-heart surgery patients, either isolated CABG, valve surgery, or combined surgery [5, 15–18]. An interesting finding from a multi-center study in Argentina stated that EuroSCORE II showed adequate performance in terms of discrimination and calibration for all types of surgery, although it was somewhat lower for coronary surgery [15]. According to H.L. Blum’s theory, the degree of a person's health can be determined by 40% of environmental factors, 30% of behavioral factors, 20% of health care factors, and 10% of genetic factors [19]. In addition, there are several factors other than health services that may be the reason for the differences in the validity of EuroSCORE II in Indonesia and abroad.

There may be some possible limitations in this study. This study was conducted at a national heart center where many patients were referred by the smaller hospital and tend to have other comorbidity that could worsen patient’s outcome. It should be noted that in this study several variables were not taken into account in EuroSCORE II, but had significant associations with mortality such as the onset of infarction and hypertension. Inaccurate patient comorbid history measured in EuroSCORE II due to patient misunderstandings during history taking can also contribute to this difference since our data based on patient anamnesis when they first brought to the ER. For example, in this study, 3.6% of patients who were not diagnosed with diabetes had HbA1C > 6.5% with 53.4% missing HbA1C data. Whereas according to research by Zheng, et.al, HbA1c levels were potentially associated with an increased risk of all-cause death, myocardial infarction, and stroke in diabetic subjects undergoing CABG surgery [20]. Research by Soewondo, et.al which examined several data sources available in Indonesia also stated that the prevalence of diabetes was 5.7%, with 70% of cases being undiagnosed [21]. Further research is needed to establish a more accurate scoring system for the Asian population, especially Indonesia, by taking into account these variables and possibly other variables that have not been taken into account in this study.

## Conclusions

Although all parameters measured in the EuroSCORE II scoring system have a significant association in increasing the risk of mortality in isolated CABG patients, the EuroSCORE II scoring system still cannot be used as an accurate predictor for the Indonesian population.

## Data Availability

The data that support the findings of this study are available from the Adult Thoracic Surgery Registry in the Research and Development Division of Harapan Kita National Cardiovascular Center, but restrictions apply to the availability of these data, which were used under license for the current study, and so are not publicly available. Data are however available from the authors upon reasonable request and with permission from the Head of Research and Development Division, Harapan Kita National Heart Center.

## Abbreviations

ACEF: Age, Creatinine, Ejection Fraction Risk Score
AUC: Area under curve
COPD: Chronic Obstructive Pulmonary Disorder
ER: Emergency Room
EuroSCORE: European System for Cardiac Operative Risk Evaluation
PCI: Percutaneous Coronary Intervention
ROC: Receiver Operating Characteristics
